# Supplementary material for: Exploring the diversity of the CO2-concentrating mechanism in different C4 subtypes
Source: J Exp Bot. 2026 Mar 3;77(12):3819–36. doi: 10.1093/jxb/erag116 (PMC13293117; doi:10.1093/jxb/erag116)
Supplement: erag116_Supplementary_Data [file erag116_supplementary_data.zip › jexbot316207-file001.pdf]

Exploring the diversity of the CO_2-concentrating mechanism (CCM) in different C_4 subtypes

Chiara Baccolini, Hirofumi Ishihara, Regina Feil, Leonardo Perez de Souza, Saleh Alseekh, Dirk Walther, Alisdair R. Fernie, Mark Stitt, John E. Lunn, Stéphanie Arrivault

Supplementary Figures and Tables

Supplementary Figure S1. Leaf protein and chlorophyll contents in C_4 species.

Supplementary Figure S2. Flow of label in C_4 photosynthesis in different labelling scenarios.

Supplementary Figure S3. $^{13}CO_2$ pulse-labelling of photosynthetic intermediates and associated metabolites in C_4 species.

Supplementary Figure S4. $^{13}CO_2$ pulse-chase labelling of photosynthetic intermediates in C_4 species.

Supplementary Figure S5. Analysis of $^{13}CO_2$ pulse and pulse-chase labelling data to estimate C_4 pathway fluxes in C_4 species.

Supplementary Table S1. Decarboxylation activities in the species studied

Supplementary Table S2. Malate and aspartate content of leaves from C_3 and C_4 species.

Supplementary Figure S1. **Leaf protein and chlorophyll contents in C_4 species.** Protein and chlorophyll were measured in randomly selected leaf samples from the pulse and pulse-chase labelling experiments on *Zea mays*, *Setaria viridis*, *Panicum miliaceum* and *Megathyrsus maximus* presented in Figures 2 and 3, except for the *Z. mays* pulse samples which were from a previously published experiment (Medeiros *et al*., 2022). The data are presented as box plots (*n*= 4) showing the median and upper and lower quartiles, with the whiskers showing the minimum and maximum values. Significant differences between species according to one-way ANOVA followed by Tukey´s multiple comparison tests are indicated by letters. The original data are in Supplementary Dataset S1B.

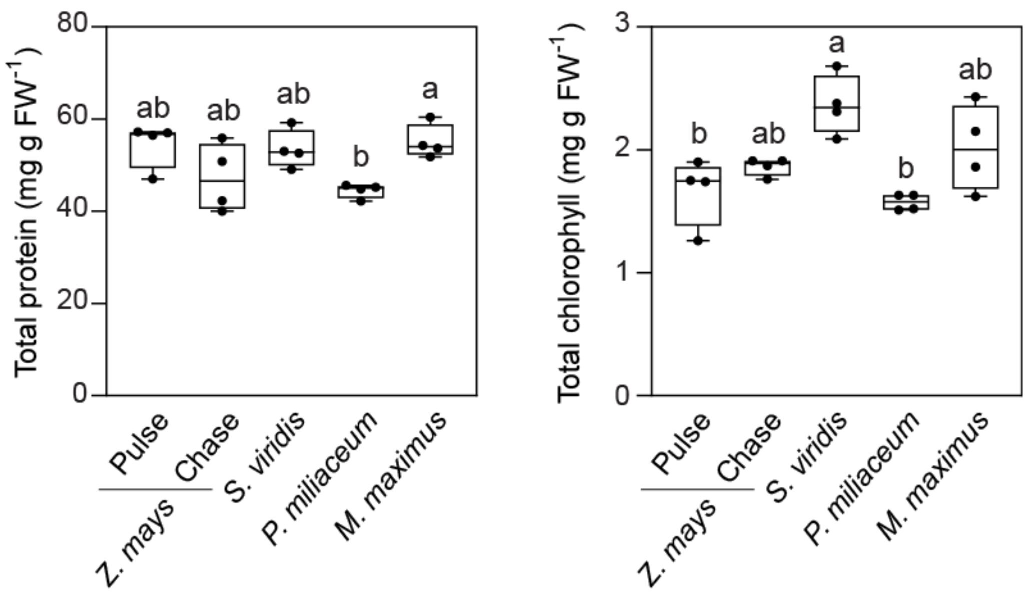

Supplementary Figure S2. Flow of label in C_4 photosynthesis in different labelling scenarios. (**A**) Labelling pattern after a short $^{13}CO_2$ pulse (≤20 s). In the mesophyll cell, PEP carboxylase incorporates ^{13}C onto the C4 position of OAA that is rapidly converted to malate or aspartate. Single labelled isotopologue (m_1) of malate and aspartate dominate the early labelling kinetics. In the bundle sheath cell, $^{13}CO_2$ is released by the decarboxylase and then re-fixed in the CBC, leading to rapid labelling of CBC intermediates, like 3PGA. Consequently, no label is introduced into the C_3 acids that return to the MC. (**B**) Labelling pattern after a short $^{13}CO_2$ pulse (20 s) followed by a $^{12}CO_2$ chase. ^{13}C is released from the C_4 acids and incorporated into the CBC intermediates, like 3PGA. (**C**) Labelling pattern after a prolonged $^{13}CO_2$ pulse. Once ^{13}C is incorporated in 3PGA, the label is randomized due to rapid turnover of the CBC. ^{13}C moves from 3PGA in the CBC to PEP in the mesophyll cell via the 3PGA:triose-phosphate shuttle that carries 3PGA to the mesophyll (grey arrow) followed by the reactions catalysed by phosphoglycerare mutase and enolase (double headed grey arrows), then from PEP into the C1-C3 position of the C_4 acids, and eventually into the C_3 acids (pyruvate and alanine, see also Arrivault *et al.*, 2017). Abbreviations: 3PGA, 3-phosphoglycerate; BSC, bundles sheath cells; CA, carbonic anhydrase; CBC, Calvin-Benson cycle; DHAP, dihydroxyacetone-phosphate; MC, mesophyll cell; OAA, oxaloacetate; PEP, phosphoenolpyruvate; PEPC, PEP carboxylase; Labelled carbon are indicated as red circles, and unlabelled carbon by white circles.

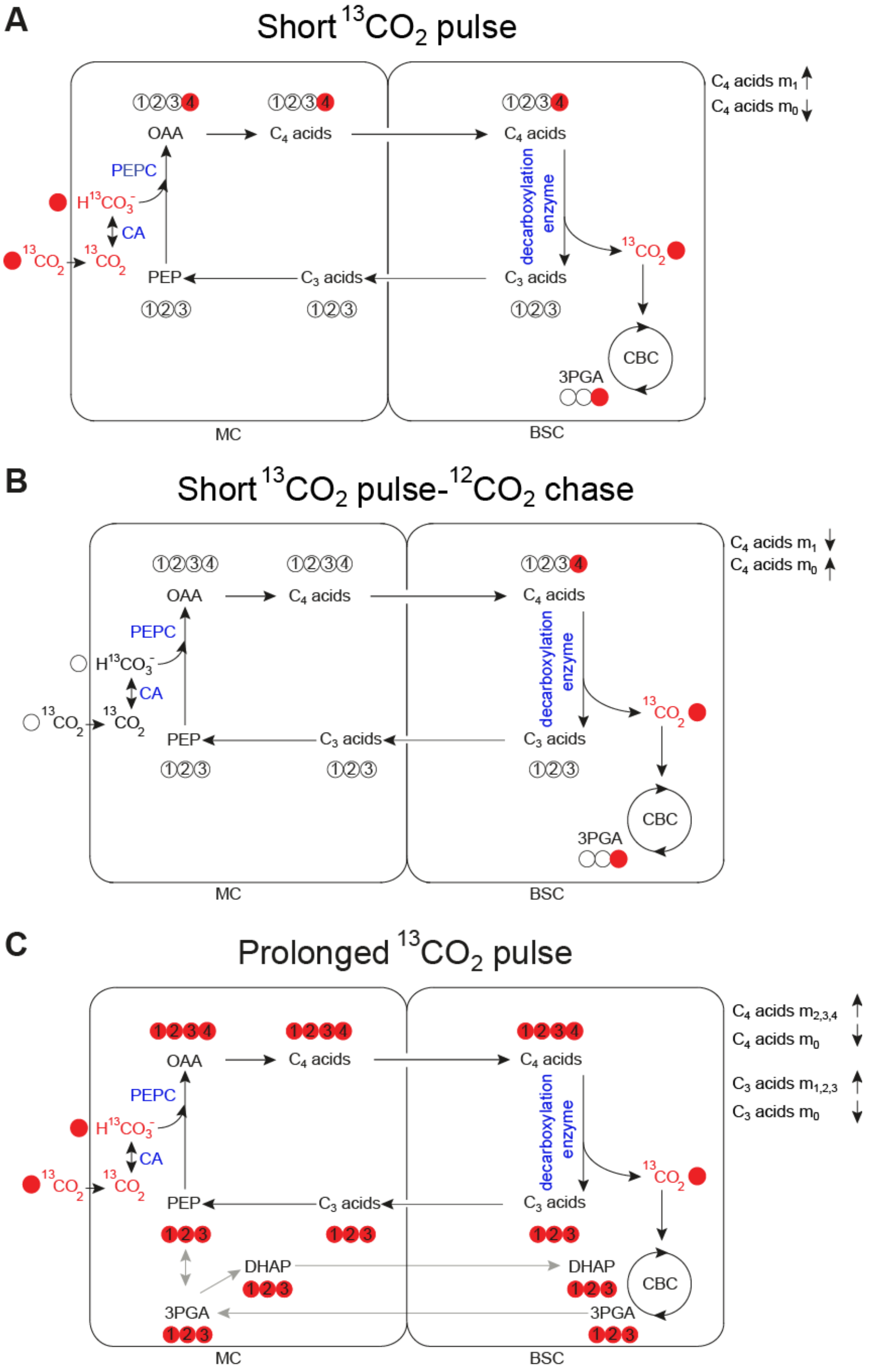

Supplementary Figure S3. **$^{13}CO_2$ pulse-labelling of photosynthetic intermediates and associated metabolites in C_4 species.** *Zea mays, Setaria viridis* (NADP-ME subtype), *Panicum miliaceum* (NAD-ME subtype) and *Megathyrsus maximus* (PEPCK subtype) leaves were pulse labelled with $^{13}CO_2$ under steady state photosynthetic conditions as described in Fig. 2. Data for *Z. mays* are from a previous study (Medeiros *et al*., 2022). The relative abundance of each isotopologue (m_x) is shown as a percentage of the total abundance of the metabolite, where x is the number of ^{13}C atoms present. Data are shown as the mean ± SD (n = 3-4). DHAP, dihydroxyacetone-phosphate; 2OG, 2-oxoglutarate. n.b. the time axis is shown on a log_{10} scale. The original isotopologue abundance data are in Supplementary Dataset S2C.

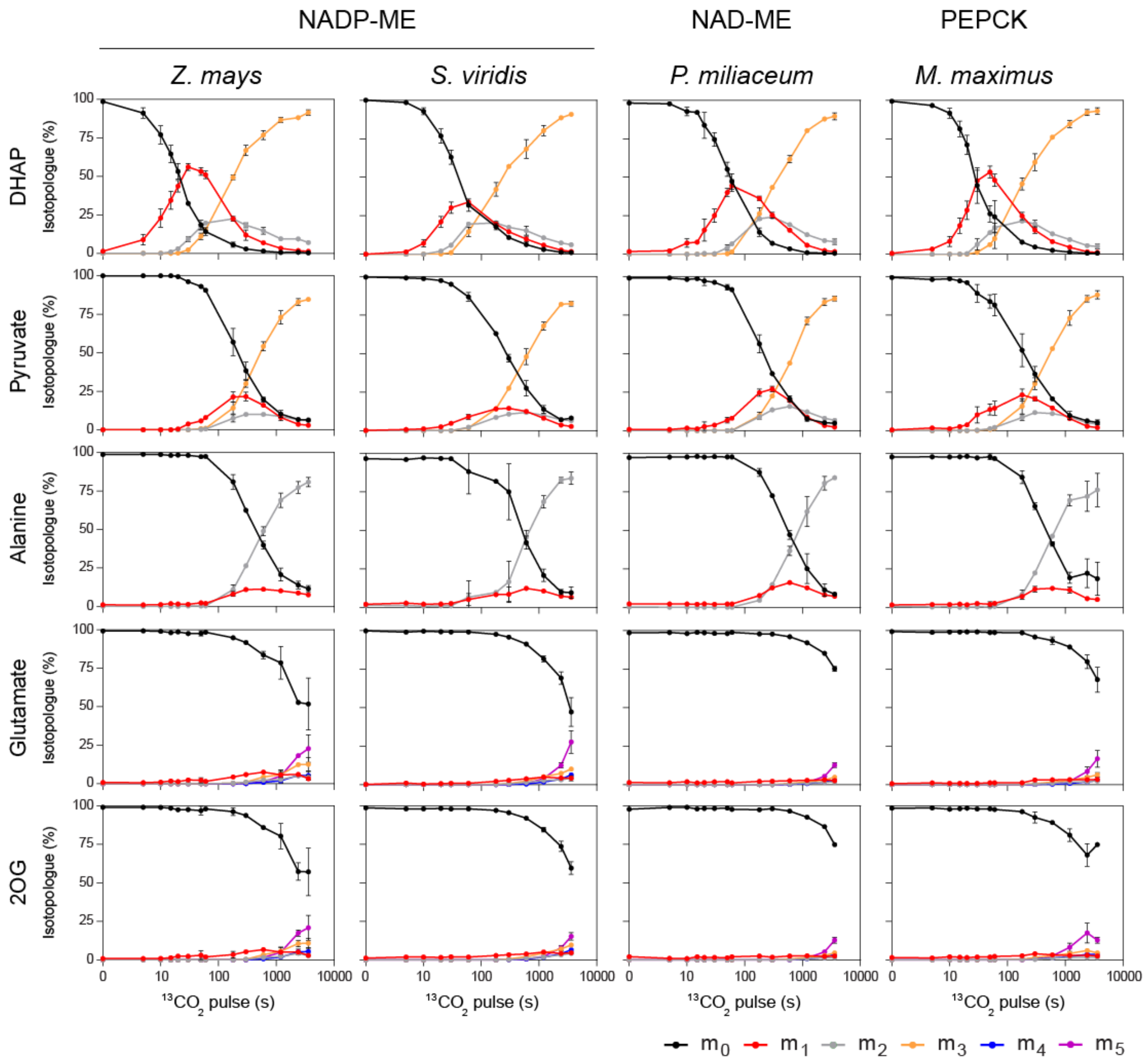

Supplementary Figure S4. **$^{13}CO_2$ pulse-chase labelling of photosynthetic intermediates in C_4 species.** *Zea mays, Setaria viridis* (NADP-ME subtype), *Panicum miliaceum* (NAD-ME subtype) and *Megathyrsus maximus* (PEPCK subtype) leaves were pulse labelled with $^{13}CO_2$ under steady state photosynthetic conditions for 20 s, followed by a chase (5-120 s) in air containing $^{12}CO_2$ as described in Fig. 3. The relative abundance of each isotopologue (m_x) is shown as a percentage of the total abundance of the metabolite, where x is the number of ^{13}C atoms present. Data are shown as the mean ± SD (n = 3-4). DHAP, dihydroxyacetone-phosphate; 2OG, 2-oxoglutarate. n.b. the time axis is shown on a log_{10} scale. The original isotopologue abundance data are in Supplementary Dataset S2C.

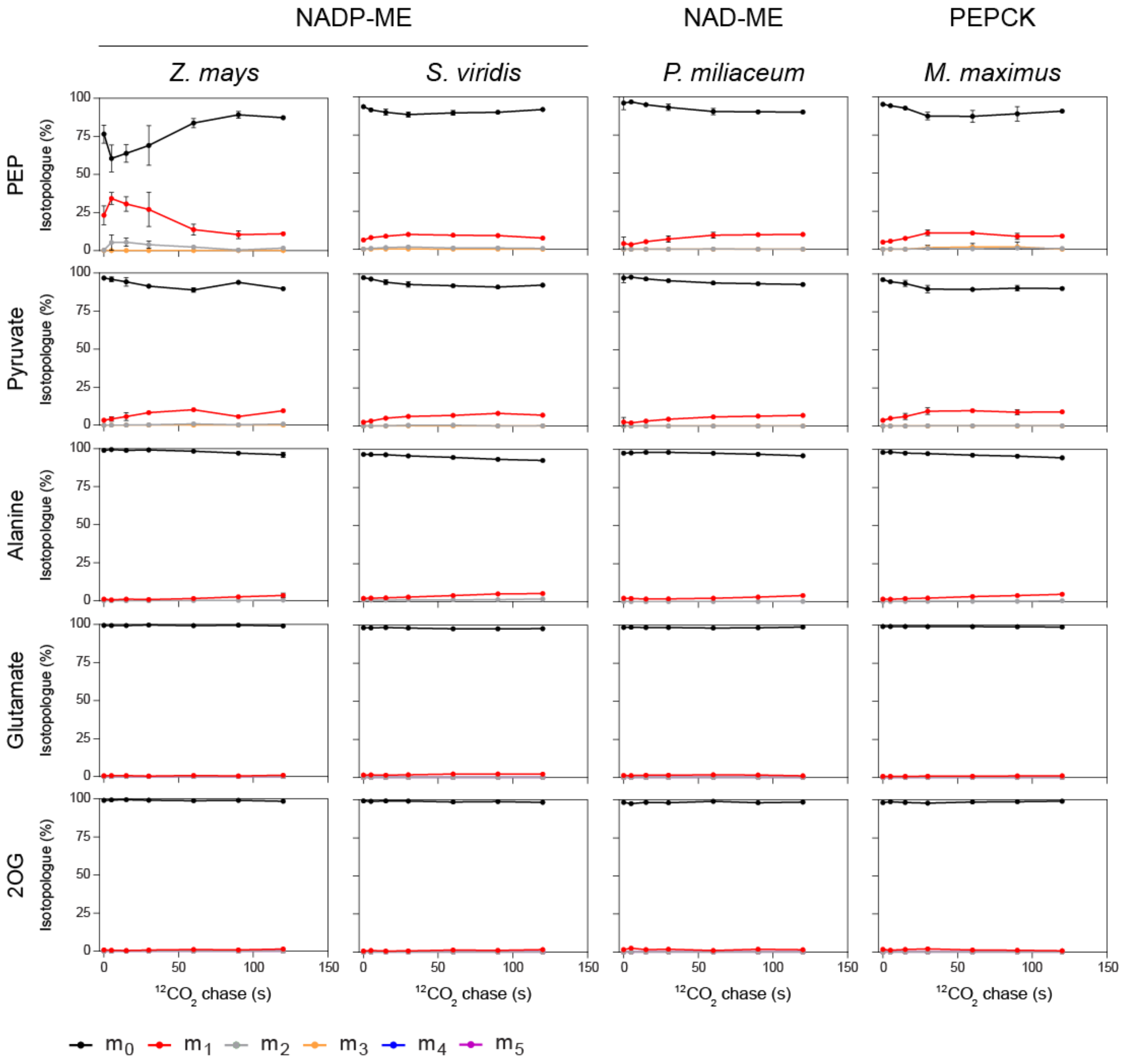

Supplementary Figure S5. **Analysis of $^{13}CO_2$ pulse and pulse-chase labelling data to estimate C_4 pathway fluxes in C_4 species.** (**A**) The fluxes of carbon into the photosynthetically active pools of malate and aspartate were estimated from changes in the abundance of the respective m_1 isotopologues during $^{13}CO_2$ pulse labelling (5-30 s; Fig. 2), serving as a proxy for specific labelling in the C4 position. Data for *Zea mays* are derived from a pulse-labelling experiment published in Medeiros *et al.* (2022). The initial carboxylation fluxes were calculated by linear regression of the data from pulse labelling times of 0-5 s. (**B**) The fluxes of carbon out of the photosynthetically active pools of malate and aspartate were estimated from changes in the abundance of the m_1 isotopologues during the $^{12}CO_2$ chase, after pulse-labelling with $^{13}CO_2$ for 20 s (Fig. 3A). For each species, a single exponential decay curve was fitted to the chase data for each metabolite (from 0-120 s), to calculate the initial rate of decarboxylation at the beginning of the chase (Supplementary Figure S5). For each species, the malate data were normalized to the respective photosynthetically active pool size (Fig. 2B) prior to analysis. The original data are in Supplementary Dataset S2F.

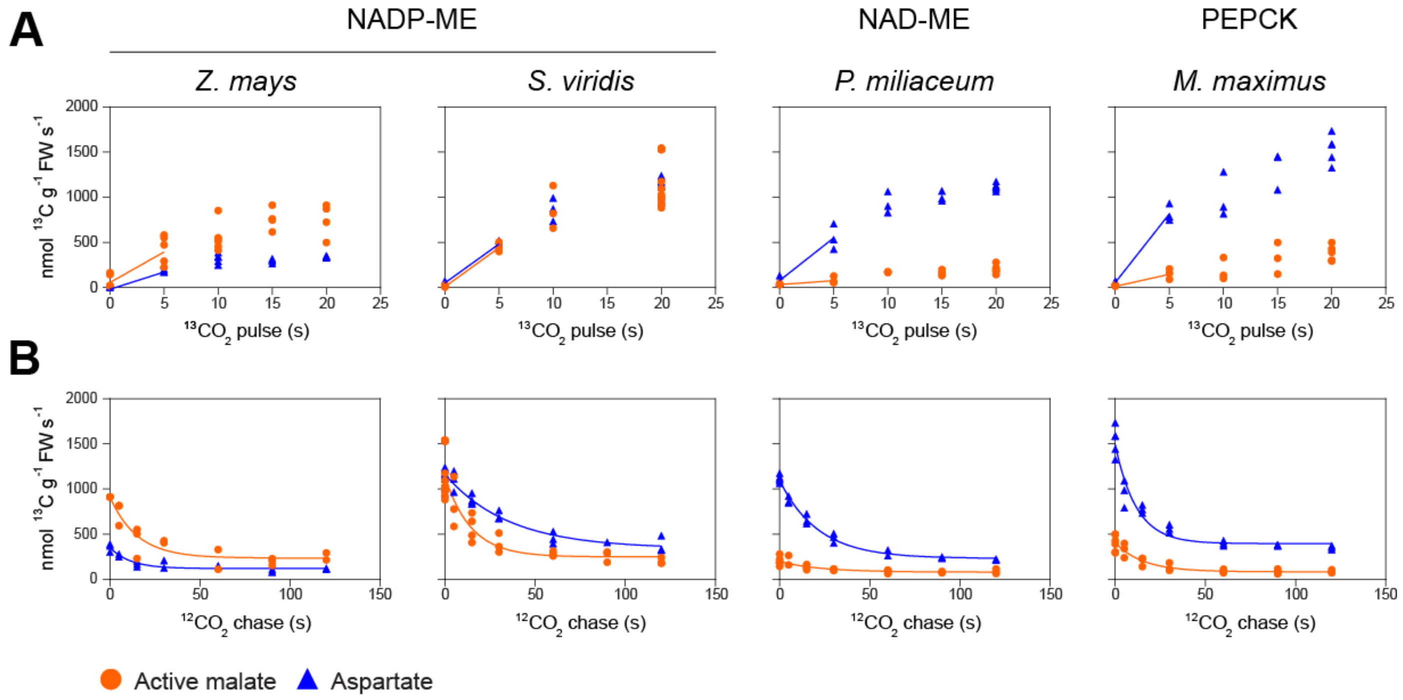

Supplementary Table S1. **Decarboxylation activities in the species studied.** Values for *Z. mays*, *P. miliaceum* and *M. maximus* are from Hatch *et al*., (1975). Values for NADP-ME activity in *S. viridis* are from Alonso-Cantabrana *et al*., (2018). The estimated values for NAD-ME are extrapolated from proteomic enrichment data (Calace *et al*. 2021). n.d. stands for not determined. Note that different units are used for *Z. mays*, *P. miliaceum* and *M. maximus*, and for *S. viridis*.

Alonso-Cantabrana H, Cousins AB, Danila F, Ryan T, Sharwood RE, von Caemmerer S, Furbank RT. 2018. Diffusion of CO_2 across the Mesophyll-Bundle Sheath Cell Interface in a C_4 Plant with Genetically Reduced PEP Carboxylase Activity. Plant Physiology **178**, 72-81. doi: 10.1104/pp.18.00618.

Calace P, Tonetti T, Margarit E, Figueroa CM, Lobertti C, Andreo CS, Gerrard Wheeler MC, Saigo M. 2021. Understanding C_4 photosynthesis in Setaria by a proteomic and kinetic approach. bioRxiv 2021.03.16.435684; doi: https://doi.org/10.1101/2021.03.16.435684

Hatch MD, Kagawa T, Craig S. 1975. Subdivision of C_4 pathway species based on differing C_4 acid decarboxylating systems and ultrastructural features. Australian Journal of Plant Physiology **2**, 111-128

Subtype	Species	Activities		
		NADP-ME	NAD-ME	PEPCK
			μmol min^{-1} mg^{-1} chlorophyll	
NADP-ME	*Zea mays*	10.7	0.23	<0.2
NAD-ME	*Panicum miliaceum*	0.4	<0.2	4.8
PEPCK	*Megathyrsus maximus*	0.2	9.8	0.5
			μmol m^{-2} s^{-1}	
NADP-ME	*Setaria viridis*	36.9 ± 2.4	4-3-6.0 (estimated)	n.d.

Supplementary Table S2. **Malate and aspartate content of leaves from C_3 and C_4 species.** The total pool sizes in C_4 species are compared with published values from C_3 species. The size of the photosynthetically active malate pool in C_4 species is also compared with the non-vacuolar pools in C_3 species where data are available. The malate data from the C_4 species show the total pools (from Figure 4) and the photosynthetically active pools determined as described in Figure 2B. Data for Spinacea oleracea are expressed in µmol g^{-1} Chlorophyll. n.a., data not available. [a]Arrivault *et al*. (2015); [b]Szecowka *et al*. (2013); [c]non-vacuolar pool; [d]Gerhardt and Heldt (1984); [e]Ermakova *et al*., 2021; [f]Medeiros *et al*. (2022).

	Species	Malate		Aspartate
		Total	Active	
		µmol g^{-1} FW	µmol g^{-1} FW	µmol g^{-1} FW
C_3	*Arabidopsis thaliana* [a]	8.64 ± 0.81	n.a.	2.10 ± 0.21
	Arabidopsis thaliana [b]	1.82 ± 0.55	0[c]	n.a.
	Nicotiana tabacum [a]	25.42 ± 1.67	n.a.	1.02 ± 0.63
	Spinacea oleracea (day)[d]	6.3	0.18[c]	n.a.
	Spinacea oleracea (night)[d]	1.8	0.05[c]	n.a.
	Triticum aestivum [a]	3.7 ± 0.25	n.a.	2.14 ± 0.14
	Oryza sativa [e]	2.56 ± 0. 89	n.a.	2.30 ± 0.34
C_4	*Zea mays* (pulse)[f]	5.64 ± 2.36	0.91 ± 0.56	0.46 ± 0.30
	Zea mays (chase)	3.66 ± 1.10	0.36 ± 0.18	0.74 ± 0.65
	Setaria viridis	3.56 ± 0.98	1.89 ± 0.76	1.98 ± 0.67
	Panicum miliaceum	1.93 ± 0.47	0.28 ± 0.24	1.67 ± 0.56
	Megathyrsus maximus	2.11 ± 0.67	0.49 ± 0.27	2.47 ± 1.14
